# Electron beam driven ion-acoustic solitary waves in plasmas with two kappa-distributed electrons

**DOI:** 10.1038/s41598-023-43422-1

**Published:** 2023-09-29

**Authors:** M. M. Hatami

**Affiliations:** Physics Department, K. N. Toosi University of Technology, Tehran, 15418-49611 Iran

**Keywords:** Space physics, Physics

## Abstract

Formation and the basic features of arbitrary amplitude ion-acoustic solitary waves (IASWs) in a plasma consisting of warm positive ions, two $\kappa$-distributed electrons and an electron beam are investigated by using the Sagdeev pseudopotential approach. It is shown that the soliton existence domain (Mach number limits) sensitively depends on temperature of ions, spectral index of cool electrons and concentration of hot electron species while spectral index of hot electrons, hot-to-cool electron temperature ratio and also concentration of electron beam do not considerably affect this domain. It is also found that temperature of electron beam only affect the existence domain of rarefactive solitons. Furthermore, it is shown that considered plasma medium supports the coexistence of positive and negative IASWs. Moreover, effect of different plasma parameters such as hot-to-cool electron density ratio, ion-to-cool electron temperature ratio, beam-to-ion density ratio, hot-to-cool electron temperature ratio and superthermality index of electron species on the basic features of positive and negative IASWs is investigated numerically. Finally, the effect of plasma parameters on the parametric regime of coexistence of compressive and rarefactive IASWs is studied and, for example, effect of temperature of positive ions and number density of hot electrons on polarity of IASWs is numerically investigated.

## Introduction

Ion-acoustic solitary waves (IASWs) are an important class of nonlinear phenomena in plasma systems. These waves which are the result of reaching the balance of nonlinear and dispersive effects can have different properties depending on the characteristics of the plasma medium. Assuming the Maxwellian distribution function being the most probable distribution function for electrons, many authors have studied the properties of these waves in different plasma systems (see, for example, Refs.^1,2^). However, observations of astrophysical and space plasmas have confirmed the existence of particle populations that are not in thermal equilibrium and so the particle distribution significantly deviates from the Maxwellian distribution due to the presence of superthermal particles having high energy tails^3–6^. These energetic particles can be described by $\kappa$-distribution rather than Maxwellian^7–10^:

1 $$\begin{aligned} f_{\kappa }(v)=\left( \frac{n_0\Gamma (\kappa )}{\Gamma (\kappa -1/2)(\pi \kappa \theta ^2)^{1/2}}\right) \left( 1+\frac{v^2}{\kappa \theta ^2}\right) ^{-\kappa }. \end{aligned}$$

Here, $\Gamma$ is the gamma function, $\kappa$ is the spectral index with $\kappa >3/2$, $n_0$ is the equilibrium density and $\theta$ is the modified thermal speed of a particle of mass $m_\alpha$ and temperature *T* given by

2 $$\begin{aligned} \theta ^2=\left( \displaystyle \frac{\kappa -3/2}{\kappa }\right) \left( \displaystyle \frac{2k_BT}{m_\alpha }\right) , \end{aligned}$$

where $k_B$ is the Boltzmann constant. It should be noted here that the $\kappa$-distribution approaches a Maxwellian distribution in the limit $\kappa \rightarrow \infty$.

Integrating the $\kappa$-distribution over velocity space, the electron number density can be written as follows:

3 $$\begin{aligned} n_{e}(\varphi )=n_{0e}\left( 1-\frac{e\varphi }{(\kappa -3/2)k_BT_e}\right) ^{-\kappa +1/2}, \end{aligned}$$

where $\varphi$ is the electrostatic potential and $n_{0e}$ and $T_e$ are the equilibrium number density and temperature of electrons, respectively.

Investigating the existence and propagation of nonlinear waves in plasmas containing two-temperature electron species (cool and hot) has received a great deal of attention. Various observations by satellite missions have confirmed the existence of such plasmas in space and astrophysical environments^11–14^. Moreover, space observations have illustrated a best fit for cool and hot electron velocity distributions with $\kappa$-distributions with low values of $\kappa$^14^. For this reason, the study of plasmas with $\kappa$-distribution has become one of the most interesting topics for authors in the last decade. For example, electron- and ion-acoustic solitary waves have been studied by Baluku et al.^10,15^ in a plasma with two-temperature $\kappa$-distributed electron. Also, Singh et al.^16^ have examined propagation of IASWs in a magnetized plasma composed of $\kappa$-distributed electrons and fluid ions with finite temperature. Modulational instability of ion-acoustic waves in an unmagnetized plasma with two-temperature $\kappa$-distributed electrons has been investigated by Alinejad et al.^17^. Moreover, Saberian et al.^18^ have studied the occurrence and propagation of large amplitude dust-acoustic solitary waves in a plasma consisting of negatively charged dust grains and electron-positron pairs with $\kappa$ distribution. In addition, a quasilinear approach for the electromagnetic cyclotron instabilities has been presented by Lazar et al.^19^ in an anisotropic bi-$\kappa$-distributed plasmas.

On the other hand, analysing data from Cassini spacecraft^11–14,20^ have also confirmed the existence of an electron beam in Saturn. Such a situation is very interesting due to the effect of presence of an electron beam on the modification of features and conditions for the existence of arbitrary amplitude solitary waves. Due to this fact, considerable attention has been attracted on investigation of solitary waves in electron beam-plasma systems^8,9,21–25^. For example, existence of arbitrary amplitude ion-acoustic solitary waves in an unmagnetized plasma consisting of ions, $\kappa$-distributed electrons and a cold electron beam has been studied by Saini and Kourakis^8^. Also, Nejoh et al. have theoretically investigated the possibility of the existence of large amplitude ion-acoustic waves under the influence of a warm electron beam in a plasma consisting of warm ions and hot isothermal electrons^21^. Moreover, using the reductive perturbation method, small amplitude IASWs have been studied by Esfandyari et al.^22^ in a collisionless plasma consisting of warm ions, hot isothermal electrons and a cold relativistic electron beam. Properties of ion- and electron-acoustic solitons have been investigated by Lakhina et al.^23^ in an unmagnetized multi-component plasma in the presence of ion and electron beams by using the Sagdeev pseudopotential technique. In addition, using a hydrodynamic model, propagation of ion-acoustic solitons has been studied by Saberian et al.^24^ in a plasma with warm ions, superthermal ($\kappa$-distributed) electrons and a cold electron beam. Propagation of electron-acoustic solitary waves has been investigated by Danehkar^25^ in a collisionless, unmagnetized plasma consisting of cool inertial background electrons, hot suprathermal electrons, stationary ions and a cool electron beam.

In most of the aforementioned studies, the examined plasma had Maxwellian electrons, or had only one species of non-Maxwellian electrons, or the presence of the electron beam was ignored in it. Whereas, as mentioned earlier, observations of space plasma have confirmed the presence of electron beam and two $\kappa$-distributed electrons in Saturn. This surprising fact has motivated us to numerically investigate the existence conditions and properties of the IASWs in a similar plasma medium with electron beam, warm positive ions and two $\kappa$-distributed electron species. Following Refs.^28,29^, the validity of describing the electron components as two fluids with respect to the ion acoustic time scale has been justified in "Appendix".

This work is arranged in four sections including the introduction as the first section. In Section "Model and basic equations", the basic equations of the considered plasma are presented and also an energy-balance equation is derived in order to analyse the nonlinear IASWs by using the Sagdeev pseudopotential approach. Moreover, limits of Mach number range in which the solitary waves can exist are investigated in this section. The structure of solitary waves and the effects of different parameters, e.g., density and temperature of electron beam, superthermality index of electron species, ratio of hot-to-cool electron density and temperature of ions on soliton amplitude will be discussed in Section "Results and discussion", and finally, a brief conclusion is presented in Section "Conclusion".

## Model and basic equations

We consider a collisionless, electropositive plasma consisting of warm fluid ions, two $\kappa$-distributed electrons with two different temperatures and an inertial electron beam with non-zero temperature. The fluid model is governed by the following one-dimensional equations for the ions

4 $$\begin{aligned}{} & {} \frac{\partial n_{i}}{\partial t}+\frac{\partial }{\partial x}( n_{i}v_{i})=0, \end{aligned}$$

5 $$\begin{aligned}{} & {} \frac{\partial v_{i}}{\partial t}+v_{i}\frac{\partial v_{i}}{\partial x} =-\frac{e}{m_i}\frac{\partial \varphi }{\partial x}-\frac{1}{m_in_i}\frac{\partial p_i}{\partial x}, \end{aligned}$$

6 $$\begin{aligned}{} & {} \frac{\partial p_{i}}{\partial t}+v_{i}\frac{\partial p_{i}}{\partial x}+3p_i\frac{\partial v_i}{\partial x}=0, \end{aligned}$$

and for the electron beam

7 $$\begin{aligned}{} & {} \frac{\partial n_{b}}{\partial t}+\frac{\partial }{\partial x}( n_{b}v_{b})=0, \end{aligned}$$

8 $$\begin{aligned}{} & {} \frac{\partial v_{b}}{\partial t}+v_b\frac{\partial v_{b}}{\partial x} =\frac{e}{m_e}\frac{\partial \varphi }{\partial x}-\frac{1}{m_en_b}\frac{\partial p_b}{\partial x}, \end{aligned}$$

9 $$\begin{aligned}{} & {} \frac{\partial p_{b}}{\partial t}+v_{b}\frac{\partial p_{b}}{\partial x}+3p_b\frac{\partial v_b}{\partial x}=0, \end{aligned}$$

where *e* is the unit electric charge, $\varphi$ is the electrostatic potential and $n_{i(b)}$, $v_{i(b)}$, $m_{i(b)}$ and $p_{i(b)}$ are density, velocity, mass and pressure of positive ions (beam), respectively.

The number density of $\kappa$-distributed electrons (cool and hot electrons) can be written as follows:

10 $$\begin{aligned} n_j=n_{0j}\left( 1-\frac{e\varphi }{k_BT_{ej}(\kappa _{j}-3/2)}\right) ^{-\kappa _j+1/2}, \end{aligned}$$

where $j=c,~h$ refer to cool and hot electron species and $\kappa _j>3/2$ is the spectral index of each electron species. Also, $T_{ej}$ and $n_{0j}$ are the temperature and density of species *j* at the sheath edge. Therefor, the charge neutrality condition demands $n_{0i}=n_{0c}+n_{0h}+n_{0b}$ where $n_{0b}$ is the unperturbed density of the beam electrons. Finally, the Poisson’s equation for such a plasma system is written as follows:

11 $$\begin{aligned} \frac{\partial ^2\varphi }{\partial x^2}=\frac{e}{\varepsilon _{0}}(n_{c}+n_{h}+n_b-n_{i}), \end{aligned}$$

where $\varepsilon _{0}$ is the electric permittivity of free space.

Normalizing Eqs. (4)–(11) by appropriate quantities, we obtain a dimensionless set of fluid equations as follows:

12 $$\begin{aligned}{} & {} \frac{\partial N_{i}}{\partial \tau }+\frac{\partial }{\partial X}( N_{i}u_{i})=0, \end{aligned}$$

13 $$\begin{aligned}{} & {} \frac{\partial u_{i}}{\partial \tau }+u_{i}\frac{\partial u_{i}}{\partial X} =-\frac{\partial \phi }{\partial X}-\frac{\sigma _i}{N_i}\frac{\partial {\tilde{P}}_i}{\partial X}, \end{aligned}$$

14 $$\begin{aligned}{} & {} \frac{\partial {\tilde{P}}_{i}}{\partial \tau }+u_{i}\frac{\partial {\tilde{P}}_{i}}{\partial X}+3{\tilde{P}}_i\frac{\partial u_i}{\partial X}=0, \end{aligned}$$

15 $$\begin{aligned}{} & {} \frac{\partial N_{b}}{\partial \tau }+\frac{\partial }{\partial X}( N_{b}u_{b})=0, \end{aligned}$$

16 $$\begin{aligned}{} & {} \frac{\partial u_{b}}{\partial \tau }+u_{b}\frac{\partial u_{b}}{\partial X} =\mu \frac{\partial \phi }{\partial X}-\mu \delta _b \frac{\sigma _b}{N_b}\frac{\partial {\tilde{P}}_b}{\partial X}, \end{aligned}$$

17 $$\begin{aligned}{} & {} \frac{\partial {\tilde{P}}_{b}}{\partial \tau }+u_{b}\frac{\partial {\tilde{P}}_{b}}{\partial X}+3{\tilde{P}}_b\frac{\partial u_b}{\partial X}=0, \end{aligned}$$

18 $$\begin{aligned}{} & {} \frac{\partial ^2\phi }{\partial X^2}=-\bigg [N_i-N_b-\delta _ c\left( 1-\frac{\phi }{(\kappa _c-\frac{3}{2})}\right) ^{-\kappa _c+1/2}-\delta _ h\left( 1-\frac{\phi }{\sigma _h(\kappa _h-\frac{3}{2})}\right) ^{-\kappa _h+1/2}\bigg ], \end{aligned}$$

where $$N_i=n_i/n_{0i}$$, $$N_{b}=n_b/n_{0i}$$, $$\phi =e\varphi /k_BT_{ec}$$, $$\sigma _i=T_{i}/T_{ec}$$, $$\sigma _b=T_{b}/T_{ec}$$, $$\sigma _h=T_{eh}/T_{ec}$$, $$\mu =m_i/m_e$$, $$u_i=v_i/c_{s}$$, $$u_b=v_b/c_{s}$$, $$X=x/\lambda _{D}$$, $$\tau =t/\omega _{pi}$$, $$\delta _b=n_{0b}/n_{0i}$$, $$\delta _h=n_{0h}/n_{0i}$$, $${\tilde{P}}_i=p_i/n_{0i}k_BT_i$$, $${\tilde{P}}_b=p_b/n_{0b}k_BT_b$$, $$c_s=(k_BT_{ec}/m_i)^{1/2}$$ and $$\lambda _{D}=(\varepsilon _{0}k_BT_{ec}/e^2n_{0i})^{1/2}$$.

To study the properties of stationary arbitrary amplitude IASWs, it is convenient to consider a stationary frame moving with a constant normalized velocity *M*, so called the Mach number. It means that we assume that all fluid variables in the evolution equations depend on a single variable $\xi =X-M\tau$. Using this transformation in Eqs. (12)–(18), we obtain the fluid basic equations of the considered plasma system in the form of a number of ordinary differential equations (ODEs) in a variable of $\xi$. Integrating these ODEs and applying appropriate boundary conditions for localized perturbations, namely, $\phi \rightarrow 0$, $u_i\rightarrow 0$, $N_i\rightarrow 1$, $N_{ec}\rightarrow \delta _c$, $N_{eh}\rightarrow \delta _h$, $N_b\rightarrow \delta _b$, $u_b\rightarrow u_{0b}$, ${\tilde{P}}_i\rightarrow 1$, ${\tilde{P}}_b\rightarrow 1$ at $\xi \rightarrow \pm \infty$, we obtain

19 $$\begin{aligned} 3\sigma _iN_i^4-(M^2+3\sigma _i-2\phi )N_i^2+M^2=0, \end{aligned}$$

for ion density and

20 $$\begin{aligned} 3(\frac{\mu \sigma _b}{\delta _b^2})N_b^4-\bigg ((u_{0b}-M)^2+3\mu \sigma _b+2\phi \bigg )N_b^2+\delta _b^2(u_{0b}-M)^2=0, \end{aligned}$$

for electron beam density.

Taking into account the boundary conditions $N_i\rightarrow 1$ and $N_b\rightarrow \delta _b$ at $\phi =0$ and the reality condition of $N_i$ and $N_b$, the acceptable solutions of Eqs. (19) and (20) are limited to the following two cases:

21 $$\begin{aligned} N_i= & {} \displaystyle \frac{1}{\sqrt{12\sigma _i}}\left\{ \left[ \left( M+\sqrt{3\sigma _i}\right) ^2-2\phi \right] ^{1/2}- \left[ \left( M-\sqrt{3\sigma _i}\right) ^2-2\phi \right] ^{1/2}\right\} , \end{aligned}$$

22 $$\begin{aligned} N_b= & {} \displaystyle \frac{\delta _b}{\sqrt{12\mu \sigma _b}}\left\{ \left[ \left( M-u_{0b}+\sqrt{3\mu \sigma _b}\right) ^2+2\mu \phi \right] ^{1/2}- \left[ \left( M-u_{0b}-\sqrt{3\mu \sigma _b}\right) ^2+2\mu \phi \right] ^{1/2}\right\} . \end{aligned}$$

From Eqs. (21) and (22), it is found that the reality condition of $N_i$ and $N_b$ limits the electrostatic potential value to become

23 $$\begin{aligned} \phi \le \frac{\left( M-\sqrt{3\sigma _i}\right) ^2}{2}, \end{aligned}$$

for positive solution and

24 $$\begin{aligned} \phi \ge -\frac{\left( M-u_{0b}-\sqrt{3\mu \sigma _b}\right) ^2}{2\mu }, \end{aligned}$$

for negative solution.

Substituting the value of $N_i$ and $N_b$ from Eqs. (21) and (22) into Eq. (19), multiplying both sides by $d\phi /d\xi$, integrating once and imposing the appropriate boundary conditions ($\phi \rightarrow 0$ and $d\phi /d\xi \rightarrow 0$ at $|\xi |\rightarrow \infty$), we obtain a single dimensionless nonlinear equation known as energy integral given by

25 $$\begin{aligned} \frac{1}{2}\left( \frac{d\phi }{d\xi }\right) ^2+V(\phi )=0, \end{aligned}$$

where $V(\phi )$, the Sagdeev pseudopotential, is given by

$$\begin{aligned} V(\phi )= & {} \delta _c\left[ 1-\left( 1-\displaystyle \frac{\phi }{\left( \kappa _c -\displaystyle \frac{3}{2}\right) }\right) ^{-\kappa _c+3/2}\right] +\delta _h\sigma _h \left[ 1-\left( 1-\displaystyle \frac{\phi }{\sigma _h\left( \kappa _h-\displaystyle \frac{3}{2}\right) }\right) ^{-\kappa _h+3/2}\right] \\{} & {} -\displaystyle \frac{\delta _b}{6\mu \sqrt{3\mu \sigma _b}} \Bigg (\left( A_1^2+2\mu \phi \right) ^{3/2} -A_1^3 - \left( A_2^2+2\mu \phi \right) ^{3/2} +A_2^3\Bigg ) \end{aligned}$$

26 $$\begin{aligned} -\displaystyle \frac{1}{6\sqrt{3\sigma }} \Bigg (\left( B_1^2-2\phi \right) ^{3/2}-B_1^3- \left( B_2^2-2\phi \right) ^{3/2}+B_2^3 \Bigg ), \end{aligned}$$

where $$A_1=\left( M-u_{0b}+\sqrt{3\mu \sigma _b}\right) ,$$ $$A_2=\left( M-u_{0b}-\sqrt{3\mu \sigma _b}\right) ,$$ $$B_1=\left( M+\sqrt{3\sigma _i}\right)$$ and $$B_2=\left( M-\sqrt{3\sigma _i}\right) .$$

As is well known, Eq. (26) has solitary wave solutions if the Sagdeev pseudopotential satisfies the following conditions^1^:

(i) $$V(\phi )=dV(\phi )/d\phi =0$$ and $$d^2V(\phi )/d\phi ^2<0$$ at $$\phi =0$$.
(ii) There exists a nonzero $\phi _m$ for which $V(\phi _m)\ge 0$.
(iii) $V(\phi )< 0$ when $0<\phi <\phi _m$ for positive solitary waves or $\phi _m<\phi <0$ for negative solitary waves, where $\phi _m$ is a maximum or a minimum value of $\phi$.

The condition $d^2V(\phi )/d\phi ^2=0$ at $\phi =0$ gives

27 $$\begin{aligned} -\delta _c\left( \frac{\kappa _c-1/2}{\kappa _c-3/2}\right) -\frac{\delta _h}{\sigma _h} \left( \frac{\kappa _h-1/2}{\kappa _h-3/2}\right) +\frac{\mu \delta _b}{(M-u_{0b})^2-3\mu \sigma _b}+\frac{1}{M^2-3\sigma _i}=0. \end{aligned}$$

Solving Eq. (27) numerically, we can determine the minimum Mach number value $(M_{min})$ for which IASWs will be formed. It is clearly seen that the minimum Mach number for a cold Maxwellian plasma in the absence of electron beam can be determined by setting $\sigma _i \rightarrow 0$, $\delta _b\rightarrow 0$ and $\kappa _{c,~h}\rightarrow \infty$ in Eq. (27). Moreover, the upper limit of Mach number for positive potential structures can be found by the condition $V(\phi _{max})=0$, where $\phi _{max}$ is the maximum value of $\phi$ for which the ion density is real^24,25^. From Eq. (21), it is found that the upper possible value of Mach number for positive IASWs (say $M_{max}^+$) is calculated from the requirement $V\left( \phi =(M-\sqrt{3\sigma _i})^2/2\right) \ge 0$. On the other hand, the upper possible value of Mach number for negative IASWs (say $M_{max}^-$) arises from the requirement for the reality of beam density which can be calculated from the requirement $V\left( \phi =-(M-u_{0b}-\sqrt{3\mu \sigma _b})^2/2\mu \right) \ge 0$. Therefore, it can be concluded that the region of existence of positive IASWs is determined by the positive ions while the electron beam determines the region of existence of negative IASWs.

## Results and discussion

In this section, we are going to investigate the effect of different plasma parameters such as spectral index of electron species ($\kappa _c$ and $\kappa _h$), temperature of ions, hot electron species and electron beam (via $\sigma _i$, $\sigma _h$ and $\sigma _b$) and concentration of the electron beam and hot electron species (via $\delta _b$ and $\delta _h$) on the existence and structure of arbitrary amplitude IASWs in an unmagnetized, collisionless plasma consisting of warm ions, warm electron beam and two $\kappa$-distributed electrons. Before examining the obtained results, it is appropriate to investigate the dependence of the upper and lower allowable limits of the soliton speed (Mach number) on different plasma physical parameters.

**Figure 1:**
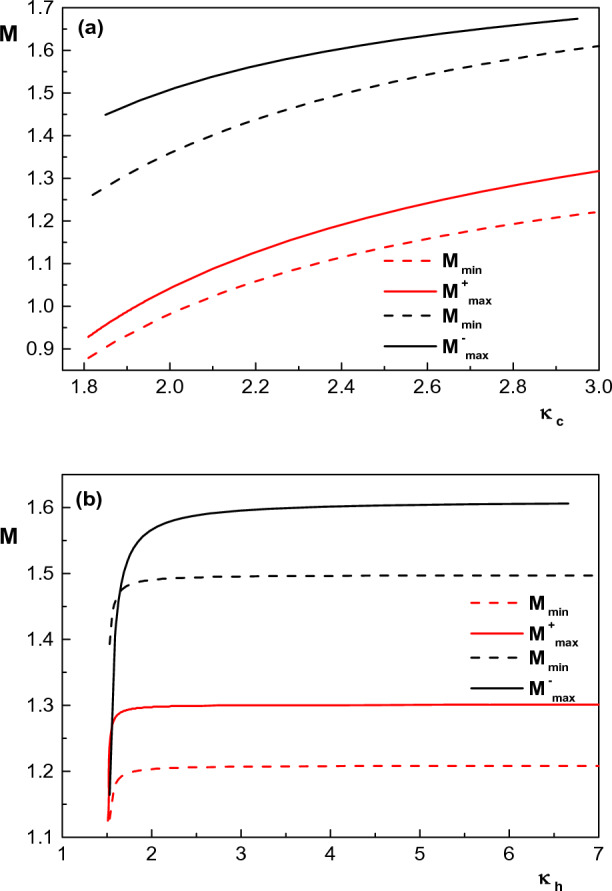
Variation of lower and upper limits of Mach number with (**a**) spectral index of cool electrons $\kappa _c$ ($1.8<\kappa _c<3$) for $\kappa _h=5$, $\sigma _i=0.1$, $\delta _h =0.5$ (red curves) and $\kappa _h=5$, $\sigma _i=0.3$, $\delta _h =0.65$ (black curves), and (**b**) spectral index of hot electrons $\kappa _h$ ($3<\kappa _h<7$) for $\kappa _c=2.9$, $\sigma _i=0.1$, $\delta _h=0.5$ (red curves) and $\kappa _c=2.4$, $\sigma _i=0.3$, $\delta _h=0.65$ (black curves). Other parameters are $u_{0b}= 0.05$, $\mu =1836$, $\sigma _b=1$, $\sigma _h=100$ and $\delta _b=0.001$.

Figure 1 shows the effect of spectral index of both cool and hot electron species ($\kappa _c$ and $\kappa _h$) on the upper and lower limit of Mach number for both positive and negative solitary waves. As we know, the requirement for the existence of IASWs is that the Mach number lies in the range of $M_{min}<M<M_{max}$. From analysing Cassini spacecraft data^14^, it has been found that the cool electrons typically have $\kappa _c\simeq 1.8-3$ in the inner magnetosphere while for the hot electrons, $\kappa _h$ typically lies in the range $3-7$. Using this fact, it can be seen from Fig. 1 that the increase in the superthemality of both cool and hot electron species, which corresponds to more energetic particles, will lower the Mach number. Also, taking into account the typical range of $\kappa _c$ and $\kappa _h$, it is seen that for $\kappa _h>3$ both upper and lower limits of Mach number have infinitesimally dependence on the hot electron spectral index. Furthermore, allowed region of compressive (rarefactive) soliton speed (region between the solid and dashed curves) increases (decreases) with increase in $\kappa _c$.

**Figure 2:**
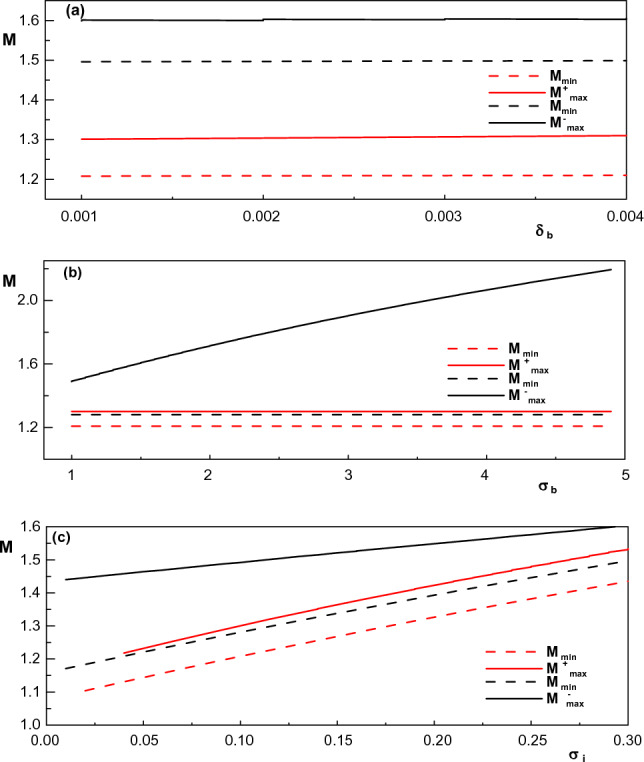
Variation of lower and upper limits of Mach number with (**a**) concentration of electron beam for $\kappa _h=5$, $\kappa _c=2.9$, $\sigma _i=0.1$, $\delta _h =0.5$ (red curves) and $\kappa _h=4$, $\kappa _c=2.4$, $\sigma _i=0.3$, $\delta _h =0.65$ (black curves), (**b**) temperature of electron beam for $\kappa _h=5$, $\kappa _c=2.9$, $\sigma _i=0.1$, $\delta _h =0.5$ (red curves) and $\kappa _h=5$, $\kappa _c=2.4$, $\sigma _i=0.1$, $\delta _h =0.65$ (black curves) and (**c**) temperature of positive ions for $\kappa _h=5$, $\kappa _c=2.9$, $\delta _h =0.5$ (red curves) and $\kappa _h=5$, $\kappa _c=2.4$, $\delta _h =0.65$ (black curves). Other parameters are $u_{0b}= 0.05$, $\mu =1836$, $\sigma _b=1$ (for (**a**) and (**c**)), $\sigma _h=100$ and $\delta _b=0.001$ (for (**b**) and (**c**)).

From Fig. 2, effect of presence of electron beam on the limits of Mach number is illustrated by examining the effect of density ($\delta _b$) and temperature ($\sigma _b$) of the electron beam on these limits. As it is seen, both $M_{max}$ and $M_{min}$ are not affected by increasing $\delta _b$ in both positive and negative potential structures. Contrary to the maximum Mach speed for existing positive IASWs $(M_{max}^+)$ , it is observed that an increase in temperature of electron beam leads to an increase in the maximum Mach number for negative IASWs $(M_{max}^-)$. As a result, the allowed range of the Mach number for existing negative IASWs increases significantly by $\sigma _b$, but it has not any significant effect on the allowed range of *M* for existing positive IASWs.

Effect of temperatures of ions ($\sigma _i$) on the upper and lower limits of *M* is also shown in Fig. 2. It is seen that for both compressive and rarefactive IASWs, both $M_{max}$ and $M_{min}$ increases by increasing the temperature of ions. Another important result has been found from Fig. 2c is that by increasing $\sigma _i$ the allowed region of soliton speed for existing rarefactive solitons reduces significantly.

**Figure 3:**
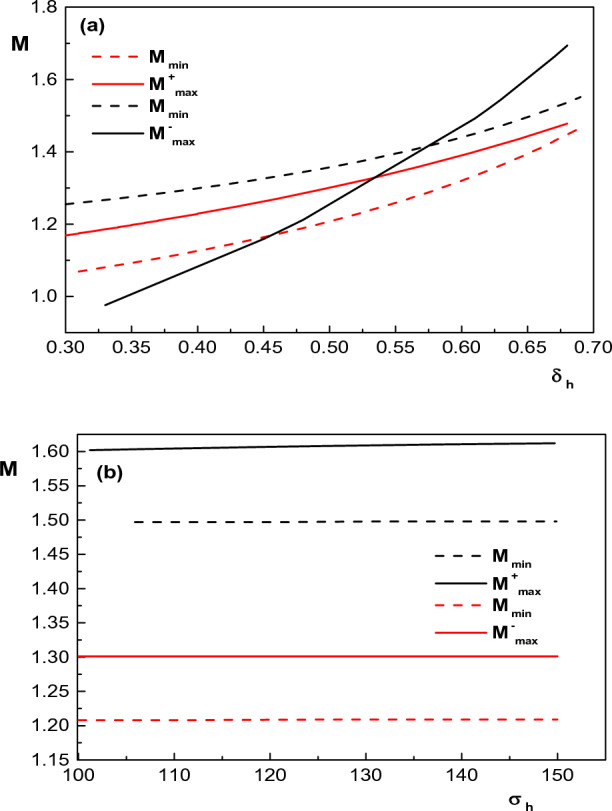
Variation of lower and upper limits of Mach number with (**a**) concentration of hot electrons for $\kappa _h=5$, $\kappa _c=2.9$, $\sigma _i=0.1$ (red curves) and $\kappa _h=4$, $\kappa _c=2.4$, $\sigma _i=0.3$ (black curves) and (**b**) temperature of hot electrons for $\kappa _h=5$, $\kappa _c=2.9$, $\sigma _i=0.1$, $\delta _h =0.5$ (red curves) and $\kappa _h=5$, $\kappa _c=2.4$, $\sigma _i=0.3$, $\delta _h =0.65$ (black curves). Other parameters are $u_{0b}= 0.05$, $\mu =1836$, $\sigma _b=1$, $\delta _b=0.001$ and $\sigma _h=100$ (for (**a**)).

In Fig. 3, an attempt has been made to show the effect of presence of the hot electrons and their temperatures on the allowable range of Mach number for existence of IASWs. We see that with increase in the hot electron density ($\delta _h$), the upper and lower limits of the Mach number increase, but the allowable range of Mach number for the existence of positive (negative) solitons decreases (increases) rapidly. In this case, it can be seen that the requirement of existence of IASWs $(M_{min}<M<M_{max})$ is violated for $\delta _h>~0.7$ and $\delta _h<~0.57$ for positive and negative IASWs, respectively. Therefore, for the formation of compressive and rarefactive IASWs in the considered plasma, there will be limits on the values of $\delta _h$. On the other hand, similar to Fig. 2, it can also be seen that any increase in temperature of hot electrons $\sigma _h$ does not have any considerable effects on the upper and lower limits of Mach number for both positive and negative potential IASWs.

**Figure 4:**
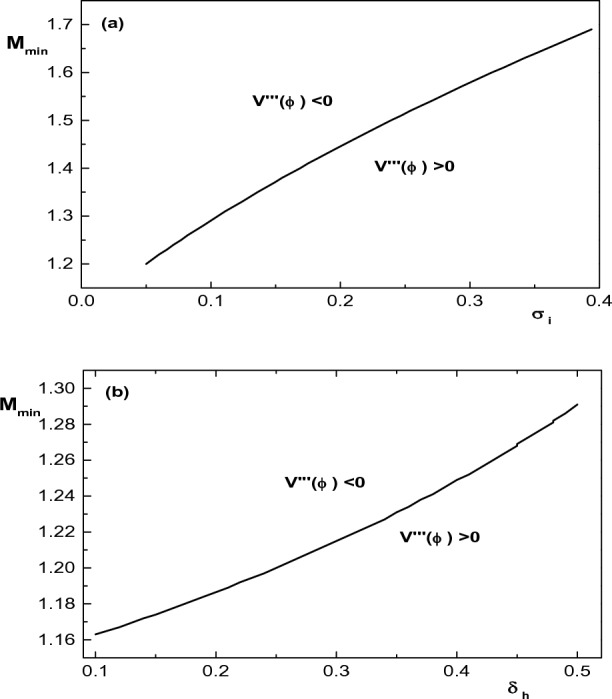
Existence domain of $M_{min}$ with (**a**) $\sigma _i$ for $\delta _h=0.5$ and (**b**) $\delta _h$ for $\sigma _i=0.1$. Below the curve, $V'''(\phi ) > 0$, and above the curve, $V'''(\phi ) < 0$. Other parameters are $\kappa _h=5$, $\kappa _c=2.9$, $u_{0b}= 0.05$, $\mu =1836$, $\sigma _b=1$, $\delta _b=0.001$ and $\sigma _h=100$.

Now, after examining the effect of different plasma parameters on the upper and lower limits of the Mach number, we are going to investigate the possibility of propagation of IASWs in the considered plasma medium and the effect of plasma parameters on these waves. It is worthwhile to mention here that the polarity of IASWs depends on the polarity of $V^{\prime \prime \prime }(\phi =0)=\left( d^3V(\phi )/d\phi ^3\right) _{\phi =0} =0$ and any point above (below) the curve $V^{\prime \prime \prime }(\phi =0)=0$ corresponds to the existence of the negative (positive) IASWs. Therefore, $V^{\prime \prime \prime }(\phi =0)=0$ gives the boundaries separating the parametric regimes for the existence of the positive and negative IASWs. As an example, the parametric regimes for the existence of positive and negative IASWs in a plasma with two-temperature $\kappa$-distributed electrons, warm ions and warm electron beam have been found by plotting $M_{min}$ with $\sigma _i$ and $\delta _h$ in Fig. 4.

**Figure 5:**
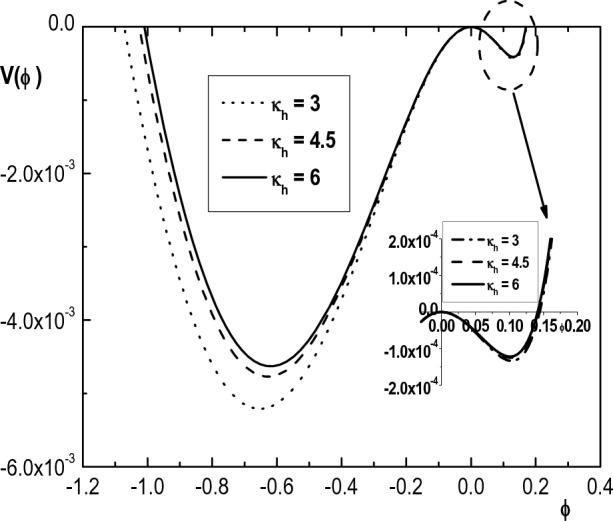
Variation of Sagdeev pseudopotential $V(\phi )$ with $\phi$ for $\kappa _c=2.4$, $u_{0b}= 0.05$, $\mu =1836$, $\sigma _b=1$, $\delta _b=0.001$, $\sigma _h=100$, $\sigma _i=0.3$, $\delta _h=0.65$, $M=1.55$ and different values of $\kappa _h$.

Figure 5 shows the variation of the pseudopotential $V(\phi )$ with the normalized potential $\phi$, for different values of the hot electron spectral index $\kappa _h$. This figure indicates that increasing $\kappa _h$ (decreasing superthermality and thus approaching the Maxwellian limit) causes the depth of pseudopotential and also the amplitude of both positive and negative IASWs to decrease. From Eq. (25), it is seen that the depth of the Sagdeev pseudopotential corresponds to the maximum value of $d\phi /d\xi$. As a result, it can be concluded that a deeper well implies a narrower soliton pulse. Therefore, the increase of superthermality will lead to the steeping of the soliton pulse. These results agree with the results of Refs.^8,10,24^. Since the effect of cool electron spectral index $\kappa _c$ on the structure of IASWs is similar to the effect of $\kappa _h$, we ignore it.

**Figure 6:**
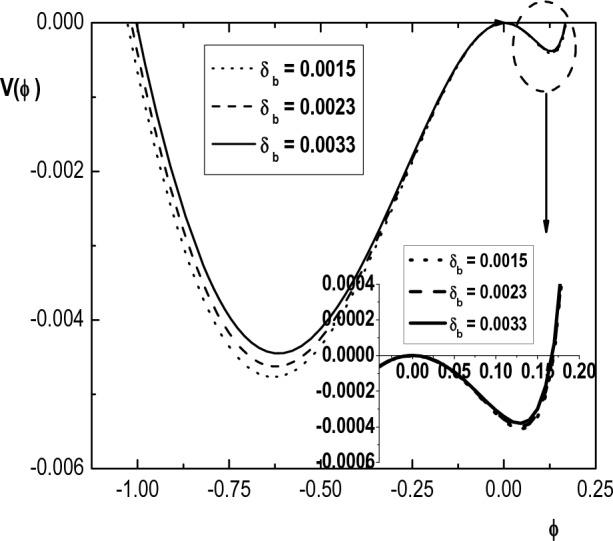
Variation of Sagdeev pseudopotential $V(\phi )$ with $\phi$ for $\kappa _h=4$, $\kappa _c=2.4$, $u_{0b}= 0.05$, $\mu =1836$, $\sigma _b=1$, $\sigma _h=100$, $\sigma _i=0.3$, $\delta _h=0.65$, $M=1.55$ and different values of $\delta _b$.

Variation of Sagdeev pseudopotential $V(\phi )$ with normalized potential $\phi$ of the compressive as well as rarefactive IASWs for different values of electron beam density (via $\delta _b$) is observed in Fig. 6. From this figure, it can be seen that the amplitude of both positive and negative solitons decreases with increase in density of electron beam. This figure also shows that the depth of the Sagdeev pseudopotential well decreases as $\delta _b$ is increased. Therefore increasing $\delta _b$ results in wider positive and negative solitons with smaller amplitude. The reason for this can be attributed to the loss of the condition for soliton formation, i.e. the balance between different contributions to the Sagdeev pseudopotential due to addition of a new component (beam electrons). However, it is observed that the change in positive soliton structure due to increasing $\delta _b$ is very smaller than that for negative one. These results agree with the result obtained in Ref.^24^ for a warm plasma with cold electron beam and one species of $\kappa$-distributed electrons.

**Figure 7:**
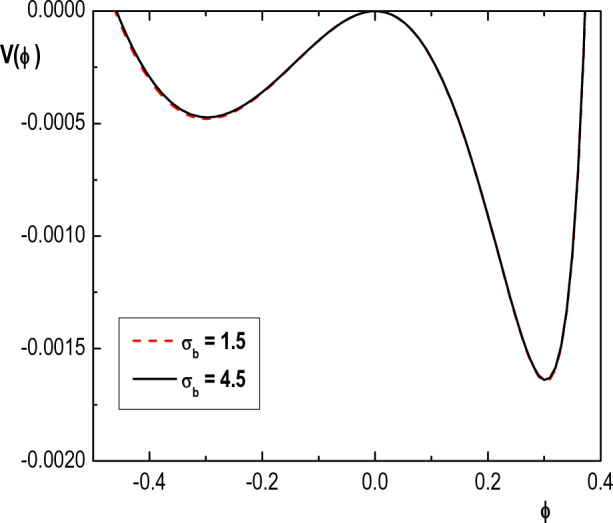
Variation of Sagdeev pseudopotential $V(\phi )$ with $\phi$ for $\kappa _h=4$, $\kappa _c=2.9$, $u_{0b}= 0.05$, $\mu =1836$, $\delta _b=0.001$, $\sigma _h=100$, $\sigma _i=0.1$, $\delta _h=0.65$, $M=1.43$ and different values of $\sigma _b$.

**Figure 8:**
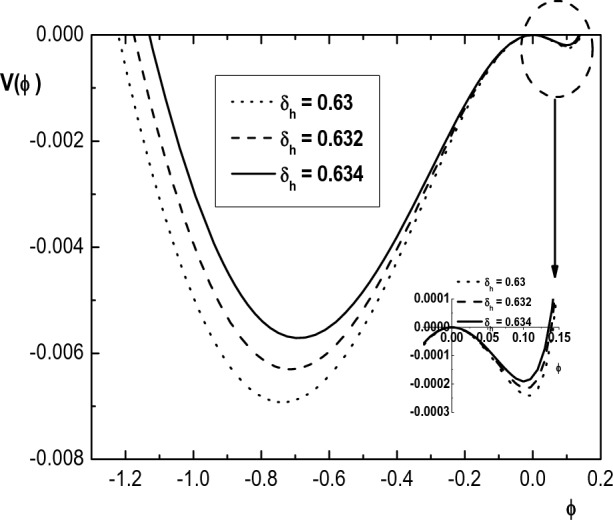
Variation of Sagdeev pseudopotential $V(\phi )$ with $\phi$ for $\kappa _h=4$, $\kappa _c=2.4$, $u_{0b}= 0.05$, $\mu =1836$, $\delta _b=0.001$, $\sigma _h=100$, $\sigma _i=0.3$, $\sigma _b=1$, $M=1.52$ and different values of $\delta _h$.

In Fig. 7, we have shown the variation of $V(\phi )$ with $\phi$ for two different values of temperature of electron beam $(\sigma _b)$. We see that the structure of Sagdeev pseudopotential and maximum amplitude of both compressive and rarefactive IASWs do not change considerably by $\sigma _b$. Therefore, it can be concluded that the properties of IASWs in a two-electron temperature plasma in Saturn do not have any considerable dependence on the temperature of electron beam.

Variation of the potential well $V(\phi )$ with $\phi$ as a result of increasing the hot electron density ($\delta _h$) is depicted in Fig. 8 for both positive and negative potential IASWs. It is seen that any increase in $\delta _h$ leads to a decrease in the width and depth of potential well which results in wider solitons with smaller maximum amplitude.

**Figure 9:**
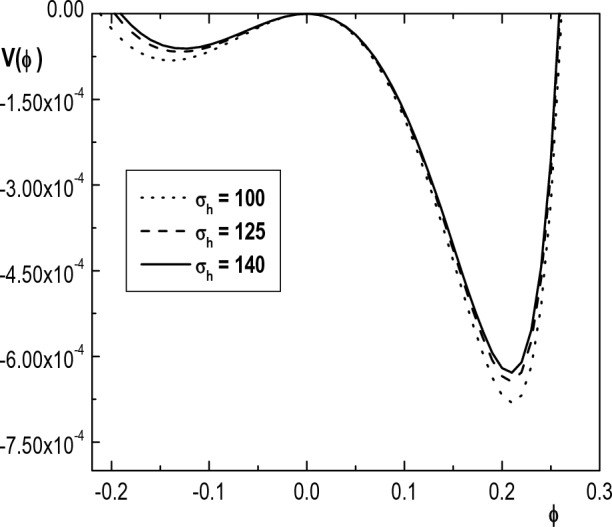
Variation of Sagdeev pseudopotential $V(\phi )$ with $\phi$ for $\kappa _h=5$, $\kappa _c=2.4$, $u_{0b}= 0.05$, $\mu =1836$, $\delta _b=0.001$, $\sigma _i=0.3$, $\sigma _b=1$, $M=1.3$ and different values of $\sigma _h$.

In Fig. 9, we have numerically shown variation of the Sagdeev pseudopotential with respect to the hot electron temperature ($\sigma _h$) in both positive and negative potential regions. We see that the depth of potential well and hence the steepness of the soliton pulse decreases by increasing the hot electron temperature. Also, similar to the result of Lakhina et al. work^26^, it is seen that the maximum amplitude of solitons decreases with an increase in temperature of hot electron species. It means that positive and negative solitons become wider as $\sigma _h$ increases.

**Figure 10:**
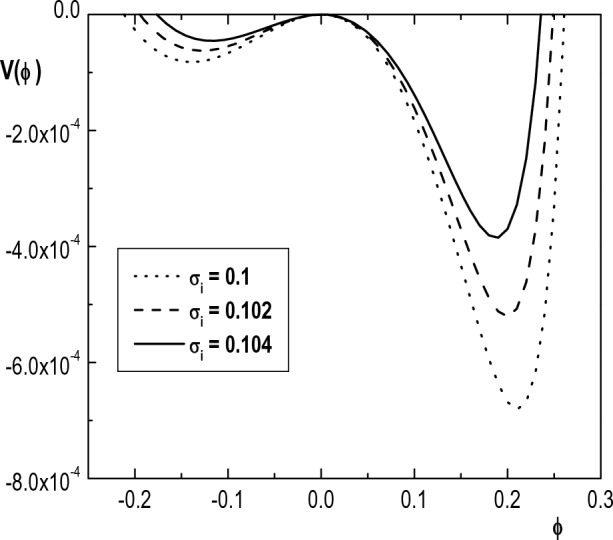
Variation of Sagdeev pseudopotential $V(\phi )$ with $\phi$ for $\kappa _h=5$, $\kappa _c=2.4$, $u_{0b}= 0.05$, $\mu =1836$, $\delta _b=0.001$, $\sigma _h=100$, $\sigma _b=1$, $\delta _h=0.65$ $M=1.3$ and different values of $\sigma _i$.

Figure 10 depicts variation of the Sagdeev pseudopotential $V(\phi )$ versus $\phi$ for different values of the positive ion temperature ($\sigma _i$). This figure reveals that the root of $V(\phi )$ (maximum amplitude of IASWs) decreases monotonically with an increase in temperature of ions. Moreover, the depth of the Sagdeev pseudopotential well decreases with an increase in $\sigma _i$. Again, it means that positive and negative solitons become wider as $\sigma _i$ increases. The decrease in amplitude of solitons by increasing ion temperatures shows that the dispersion is enhanced due to increase in ion thermal motion. Similar results have been reported in Refs.^24,27^ for IASWs in a plasma with one species of $\kappa$-distributed electrons.

## Conclusion

Using a fluid model, formation and basic features of arbitrary amplitude IASWs in a superthermal plasma consisting of two-temperature electron species with kappa distribution, warm inertial positive ions and warm electron beam were investigated. The Sagdeev pseudopotential approach was employed to determine the permitted parametric regions where allow solitons to propagate in the considered plasma. The important results of the current investigation can be summarized as follows:

1. The lower limit of soliton speed (lower limit of Mach number, $M_{min}$) sensitively depends on superthermality of electron species ($\kappa _j~_{(j=c, h)}$), temperature of ions ($\sigma _i$) and concentration of hot electrons ($\delta _h$), and increases with the increase of $\kappa _j$, $\sigma _i$ and $\delta _h$. However, temperature and concentration of electron beam ($\sigma _b$ and $\delta _b$) and also temperature of hot electron species $(\sigma _h)$ do not have any considerable effect on $M_{min}$. Moreover, effect of superthermality of hot electrons on $M_{min}$ becomes negligible for $\kappa _h>3$.
2. The upper limits of soliton speed for positive and negative potential IASWs ($M_{max}^+$, $M_{max}^-$) increase by increasing $\kappa _j$ (decreasing superthermality of electron species), $\sigma _i$ and $\delta _h$. Moreover, $\sigma _b$ only affect $M_{max}^-$, so as $\sigma _b$ increases, the upper limit of Mach number for negative potential IASWs increases. Other plasma parameters do not affect the upper bound of soliton speed considerably.
3. The existence region of compressive IASWs $(M_{min}<M<M_{max}^+)$ characterized by positive ions, but for rarefactive IASWs, this region is specified by electron beam.
4. The coexistence of compressive and rarefactive IASWs in the considered plasma system has been confirmed.
5. Depending on the values of plasma parameters, there will be limitation on values of $\delta _h$ beyond them there will be no positive or negative potential IASWs due to violation of the requirement for the existence of IASWs ($M_{min}<M<M_{max}$).
6. The parametric regimes for the existence of both polarity IASWs has been numerically investigated by plotting the existence region of $M_{min}$ with $\sigma _i$ and $\delta _h$ and it was shown that there exists a cut off region, below the curves, $V^{'''}(\phi =0)>0$, and above the curves $V^{'''}(\phi =0)<0$ which correspond to formation region of positive and negative potential IASWs, respectively.
7. Any increase in $\kappa _j$, $\sigma _b$, $\sigma _h$, $\delta _h$ and $\sigma _i$ leads to a decrease in maximum amplitude of both compressive and rarefactive IASWs and depth of Sagdeev pseudopotential well which results in wider and shorter solitons. However, $\sigma _b$ does not affect structure of IASWs considerably.

Finally, it should be mentioned that the results of the present investigation should be useful in understanding the nonlinear features of electrostatic disturbances in plasma observed in Saturn^14,20^ in which positive ions, two kappa-distributed electrons with different temperatures and an electron beam can be the major plasma species.

### Supplementary Information

Supplementary Information.

## Data Availability

All data generated or analysed during this study are included in this published article (and its supplementary information files).
