# Supplementary material for: Electron beam driven ion-acoustic solitary waves in plasmas with two kappa-distributed electrons
Source: Sci Rep. 2023 Sep 29;13:16363. doi: 10.1038/s41598-023-43422-1 (PMC10541895; doi:10.1038/s41598-023-43422-1)
Supplement: Supplementary file 1 — Supplementary Information. [file 41598_2023_43422_MOESM1_ESM.pdf]

Appendix

According to Jones et al [28], the validity of describing the electron components as two fluids is justified on the grounds that processes which produce such electron distributions have time scales much shorter than the relevant ion time scale. Following Ref. [29], the linearized dispersion relation of the ion-acoustic waves for a plasma with warm ions and two kappa distributed electrons can be written as follows:

$$1-\frac{\omega_{pi}^2}{\omega^2-k^2v_{ti}^2}+\frac{1}{k^2\lambda_{D\kappa}^2}=0, \tag{A.1}$$

where ω is the wave frequency, k is the wave number, $v_{ti}^2=3k_BT_i/m_i$, $\omega_{pi}^2=n_{0i}e^2/\varepsilon_0 m_i$ and $\lambda_{D\kappa}^{-2}=\dfrac{e^2}{\varepsilon_0 k_B}\left[\dfrac{n_{0c}}{T_{ec}}\left(\dfrac{\kappa_c-1/2}{\kappa_c-3/2}\right)+\dfrac{n_{0h}}{T_{eh}}\left(\dfrac{\kappa_h-1/2}{\kappa_h-3/2}\right)\right]$.

Using $C_{s\kappa}^2=(1+3\sigma_{if})k_BT_{eff}/m_i$, Eq. (A.1) can be written as follows:

$$\frac{\omega^2}{k^2}=C_{s\kappa}^2\left(\frac{1+\dfrac{3\sigma_{if}}{1+3\sigma_{if}}k^2\lambda_{D\kappa}^2}{1+k^2\lambda_{D\kappa}^2}\right), \tag{A.2}$$

where $\sigma_{if}=T_i/T_{eff}$ and

$$\frac{n_{0c}+n_{0h}}{T_{eff}}=\frac{n_{0c}}{T_{ec}}\left(\frac{\kappa_c-1/2}{\kappa_c-3/2}\right)+\frac{n_{0h}}{T_{eh}}\left(\frac{\kappa_h-1/2}{\kappa_h-3/2}\right). \tag{A.3}$$

Now, for a plasma with warm ions and one species of isothermal electrons ($n_{0h}=0$, $\kappa_c\to\infty$ and $T_{ec}=T_e$), the dispersion relation (A.2) takes the following form:

$$\frac{\omega^2}{k^2}=C_{sm}^2\left(\frac{1+\dfrac{3\sigma_{if}}{1+3\sigma_{if}}k^2\lambda_{Dm}^2}{1+k^2\lambda_{Dm}^2}\right), \tag{A.4}$$

where $C_{sm}^2=(1+3\sigma_{if})k_BT_e/m_i$.

From the definition of $C_{s\kappa}$, C_{sm}, $\lambda_{D\kappa}$, λ_{Dm} and T_{eff}, we get the following two inequalities:

$$C_{sm}^2<C_{s\kappa}^2 \qquad if\ \ T_e<T_{eff}, \tag{A.5}$$

and

$$\lambda_{D\kappa}^2 < \lambda_{Dm}^2. \tag{A.6}$$

Since $T_c(= T_e) < T_h$ and $\frac{\kappa_{c,h}-1/2}{\kappa_{c,h}-3/2} > 1$ for $\kappa_{c,h} > 3/2$, condition $T_e < T_{eff}$ is always true, and the inequalities (A.5) and (A.6) hold good for any values of the parameters. From these inequalities, we get

$$\frac{\lambda_{D\kappa}^2}{C_{s\kappa}^2} < \frac{\lambda_{Dm}^2}{C_{sm}^2}, \tag{A.7}$$

or

$$\omega_p^{-2} < \omega_{pi}^{-2}, \tag{A.8}$$

where $\omega_{pi}^2 = n_{0i}e^2/\varepsilon_0 m_i$ and $\omega_p = C_{s\kappa}/\lambda_{D\kappa}$.

Inequality $\omega_p^{-1} < \omega_{pi}^{-1}$ shows the validity of describing the electrons as two different species of particles of the plasma with respect to the ion acoustic time scale.
